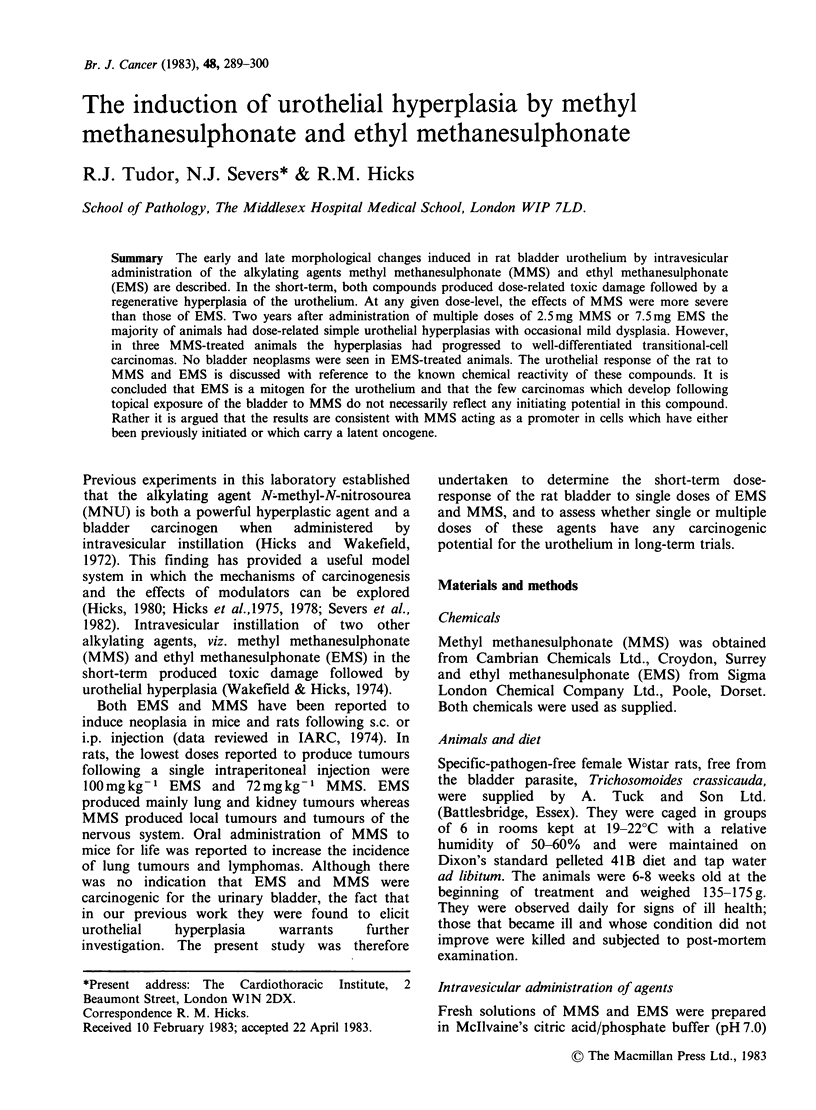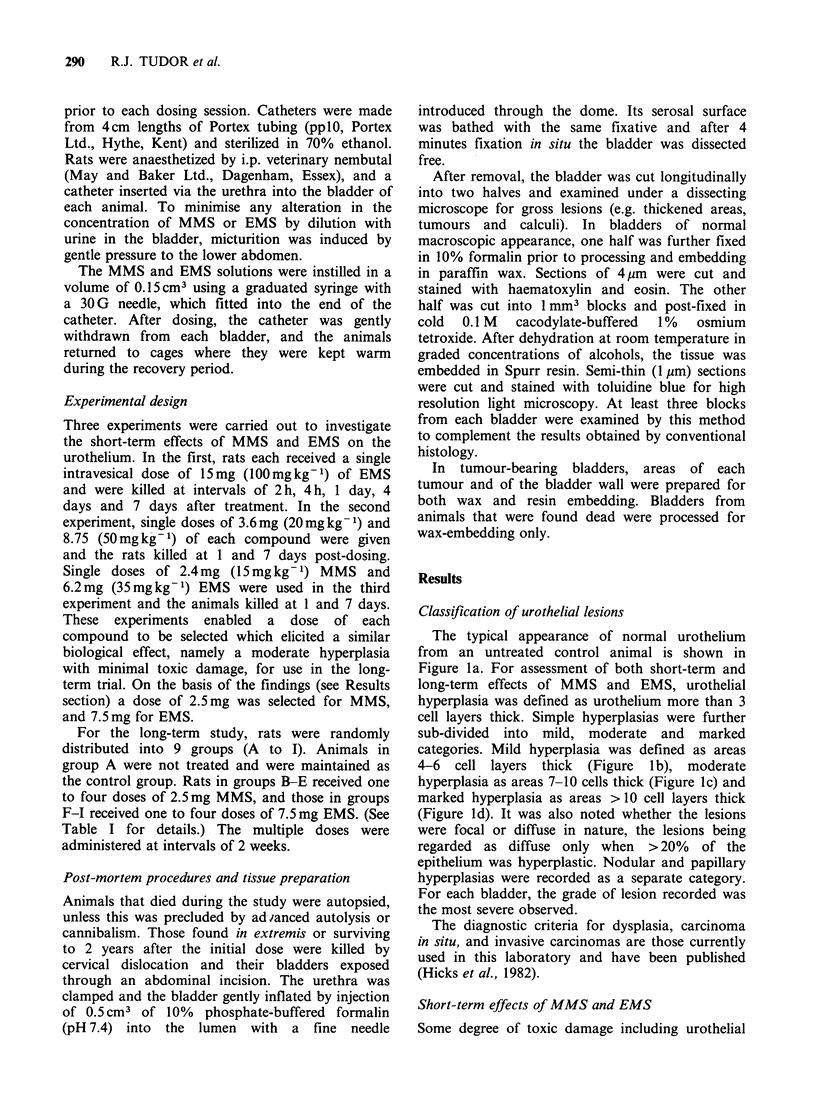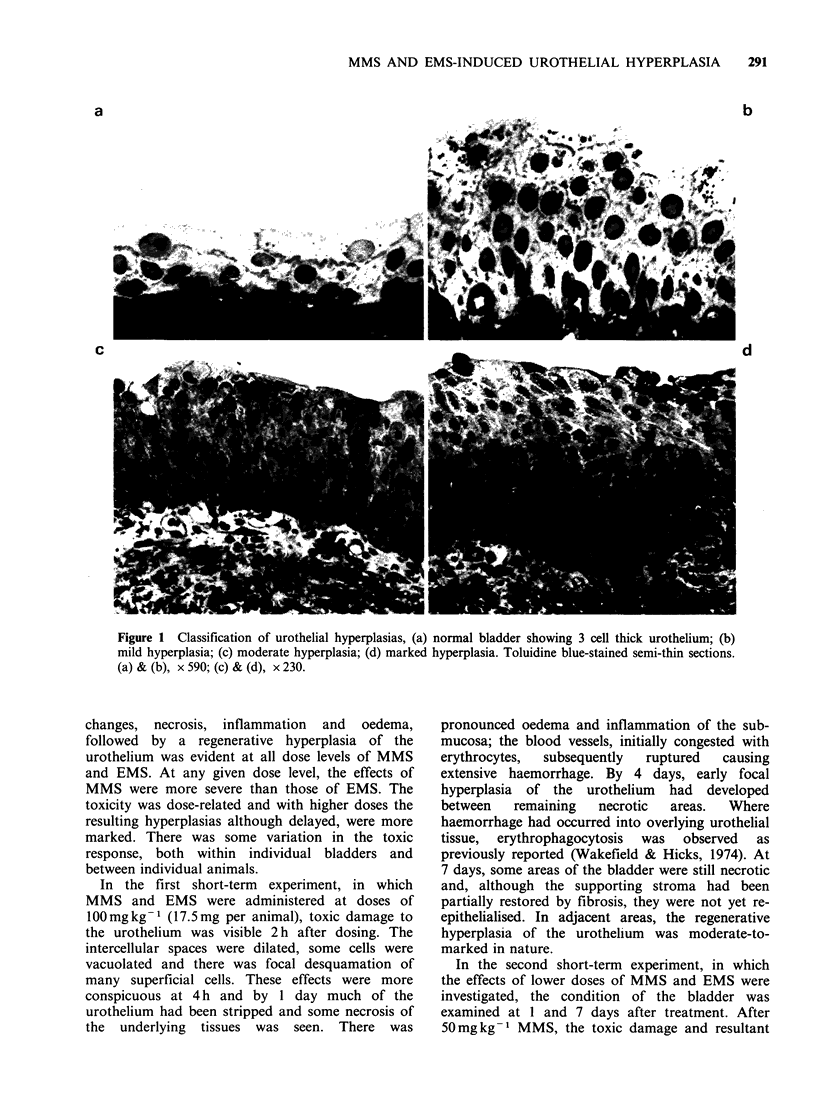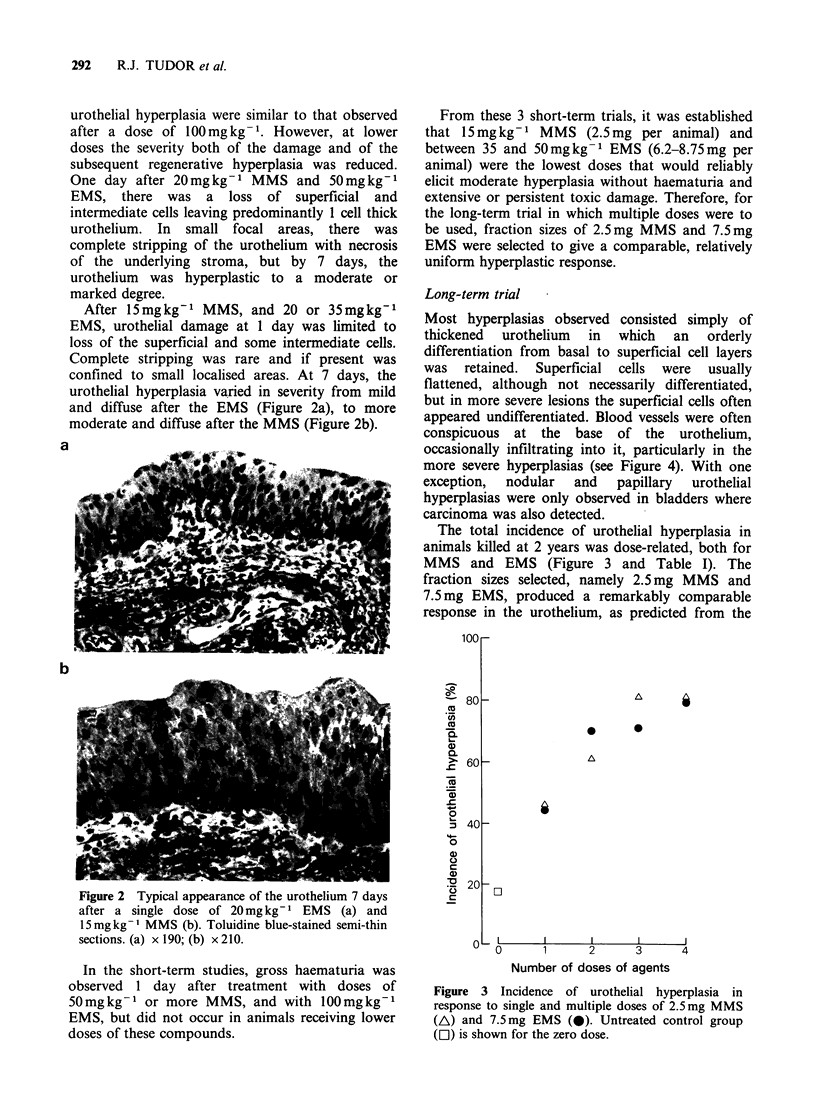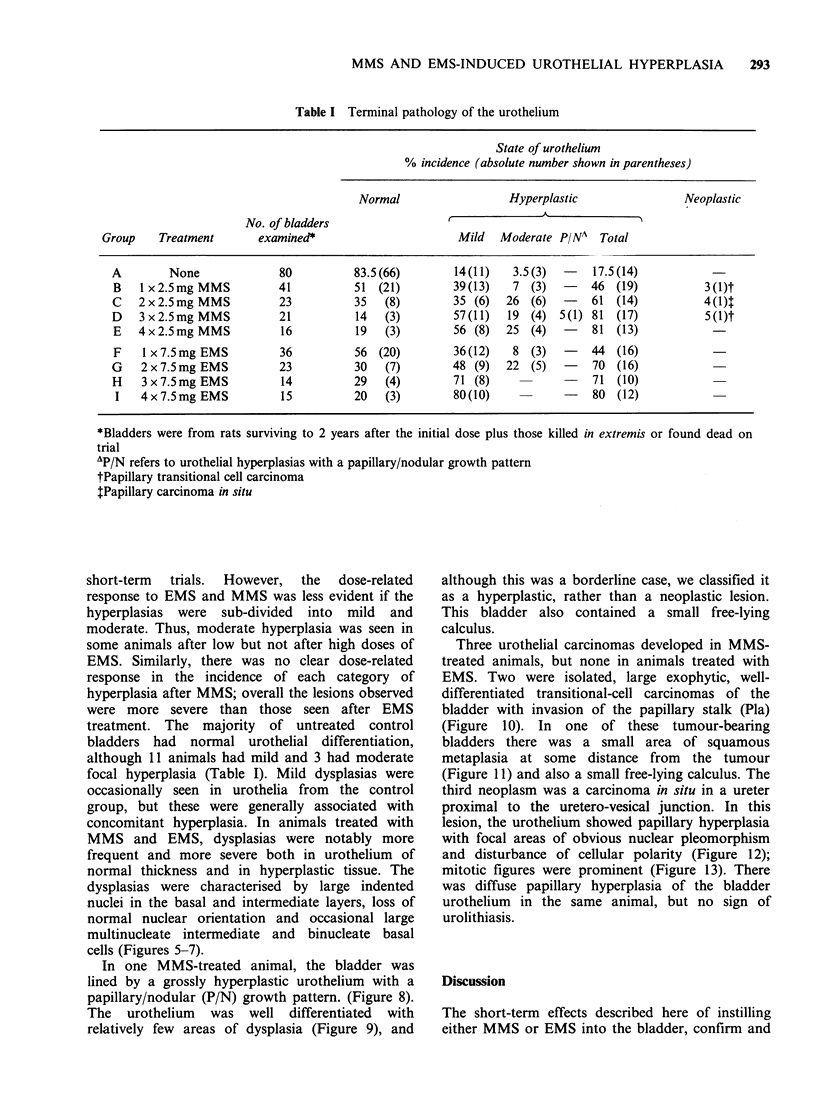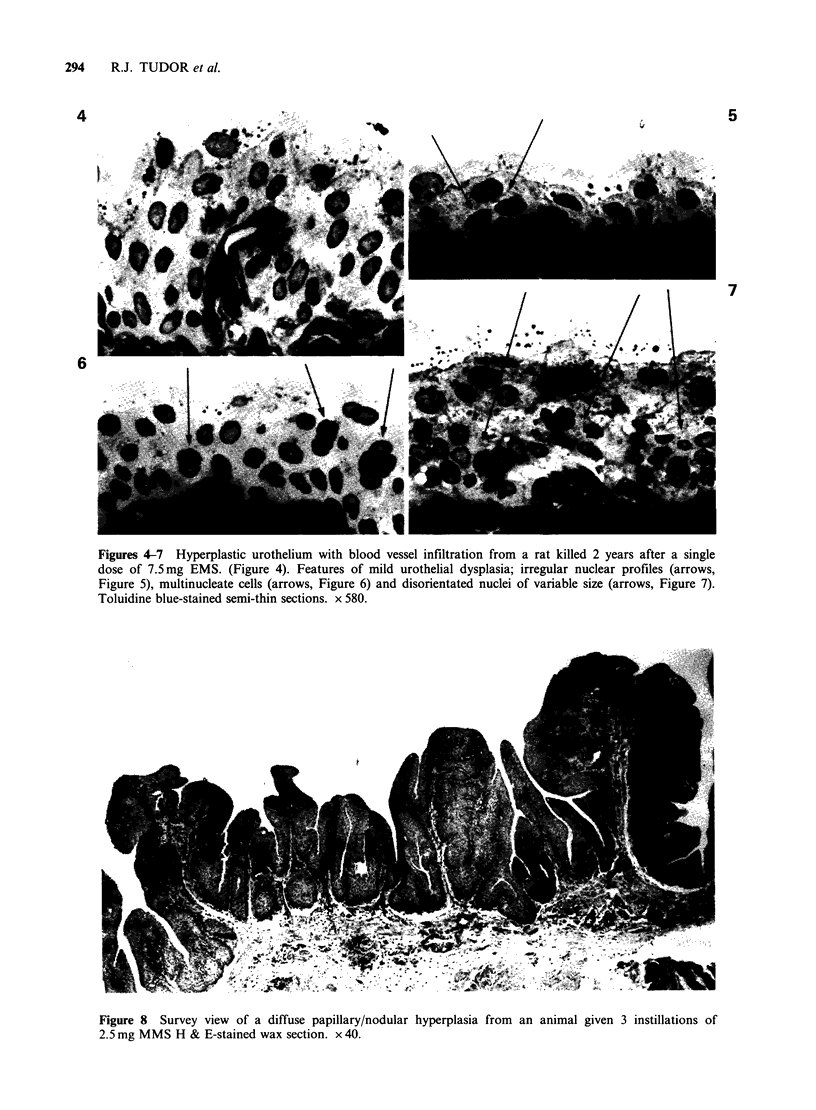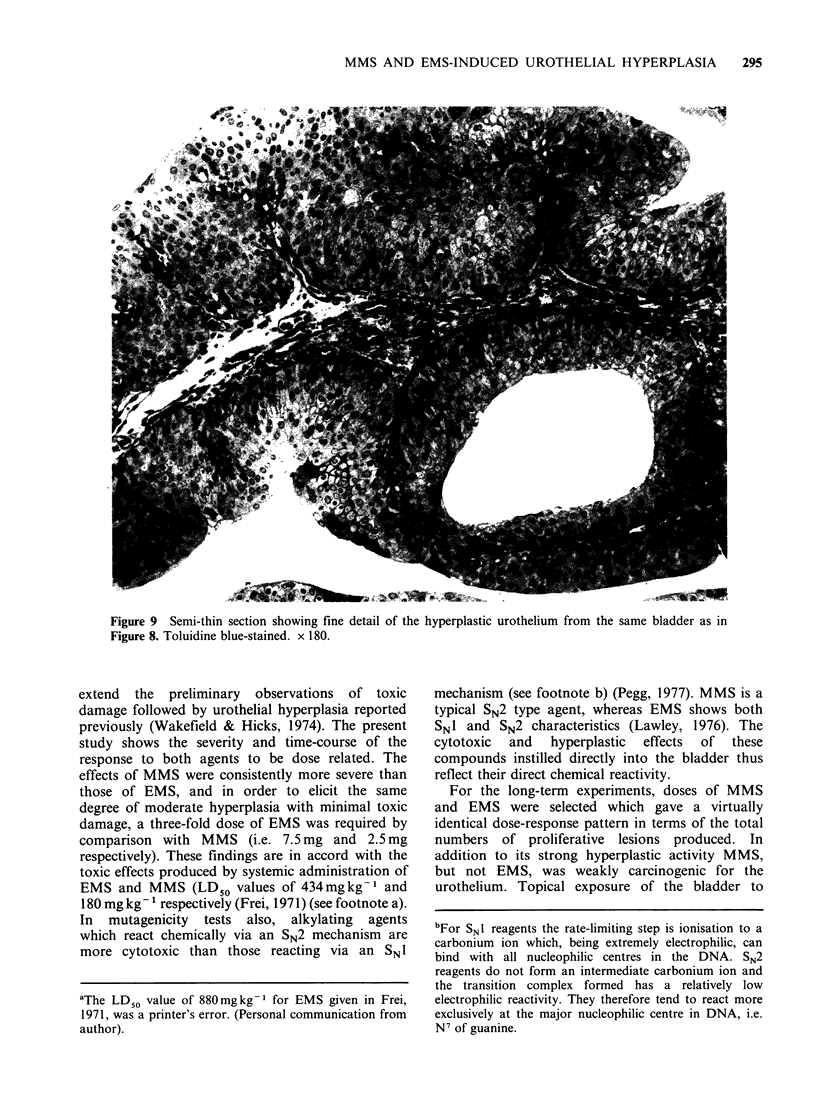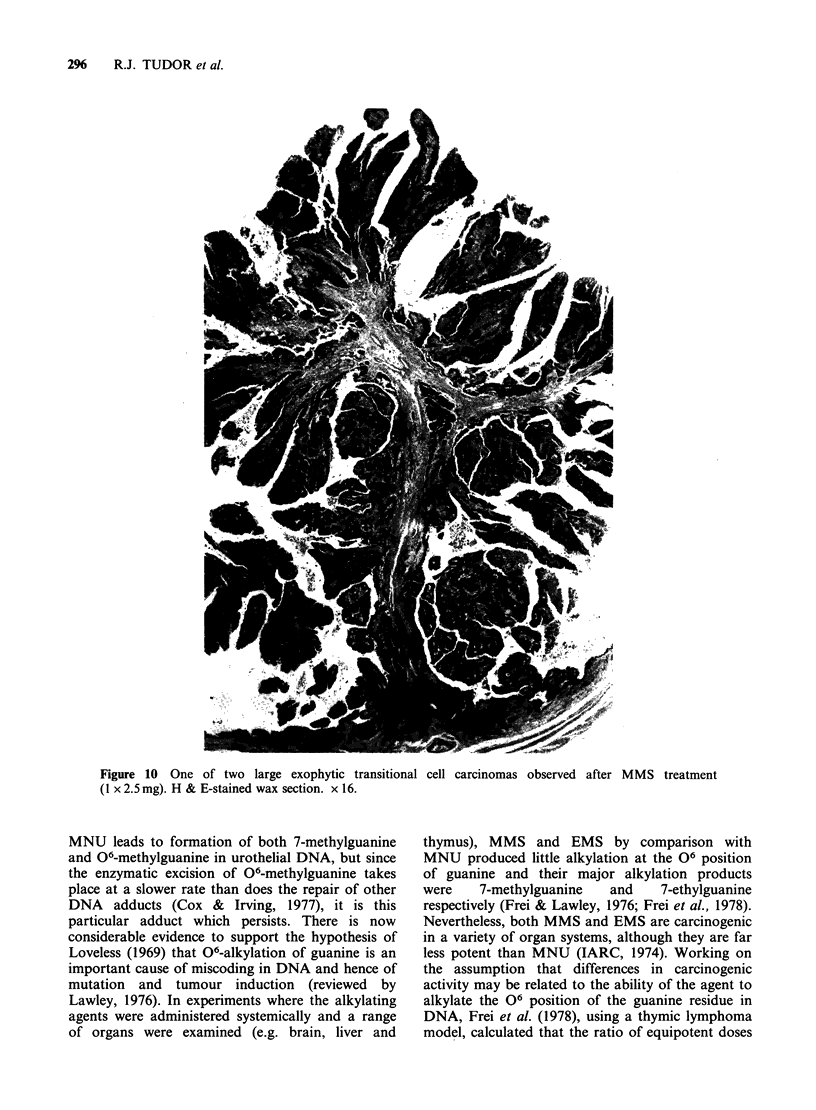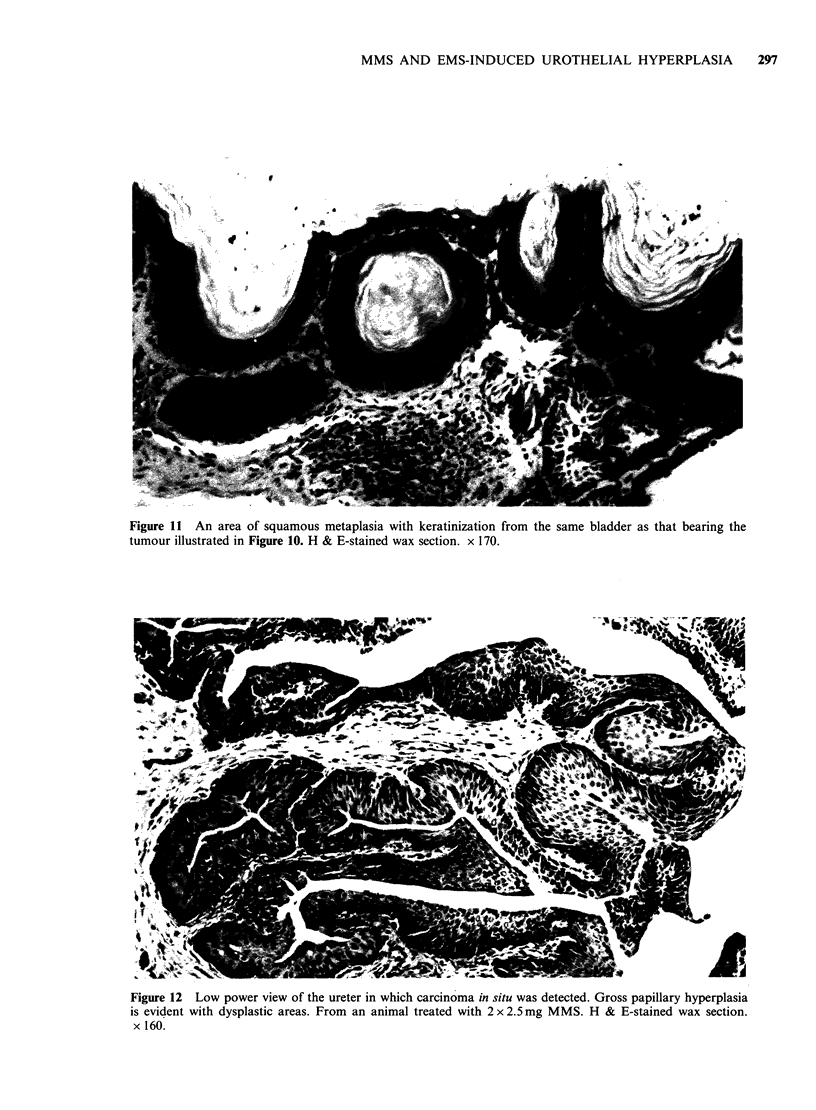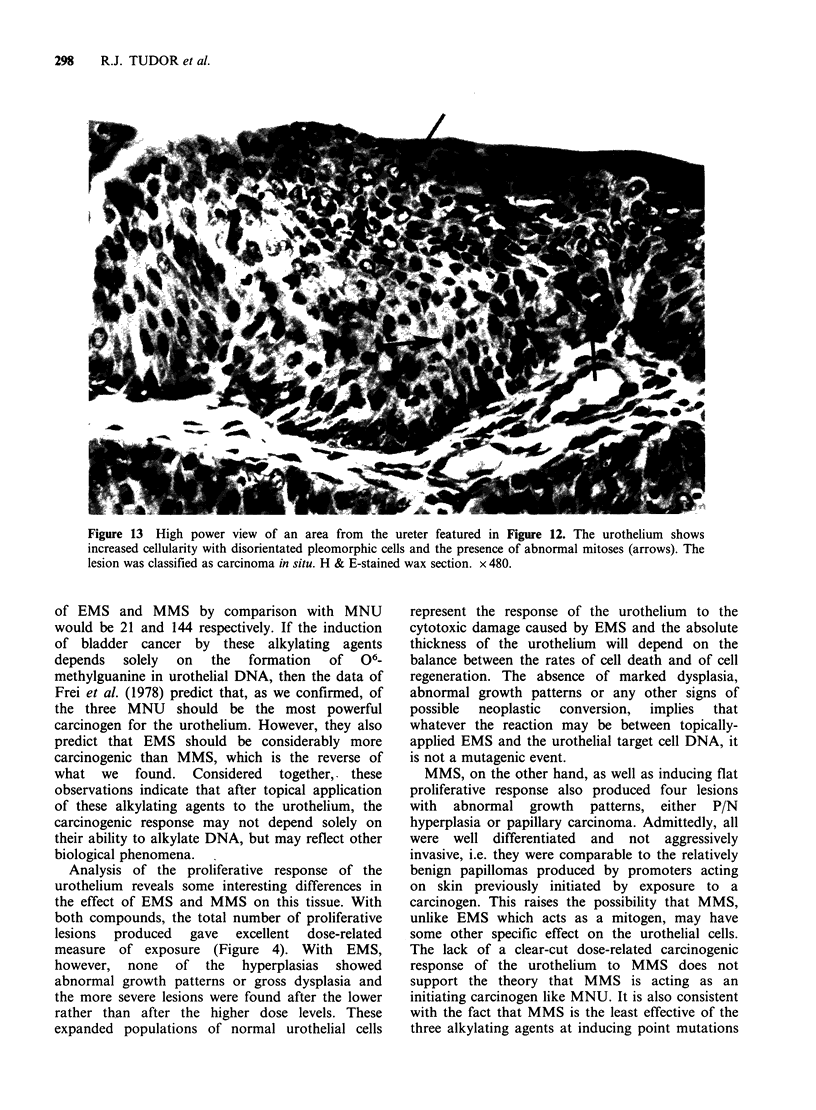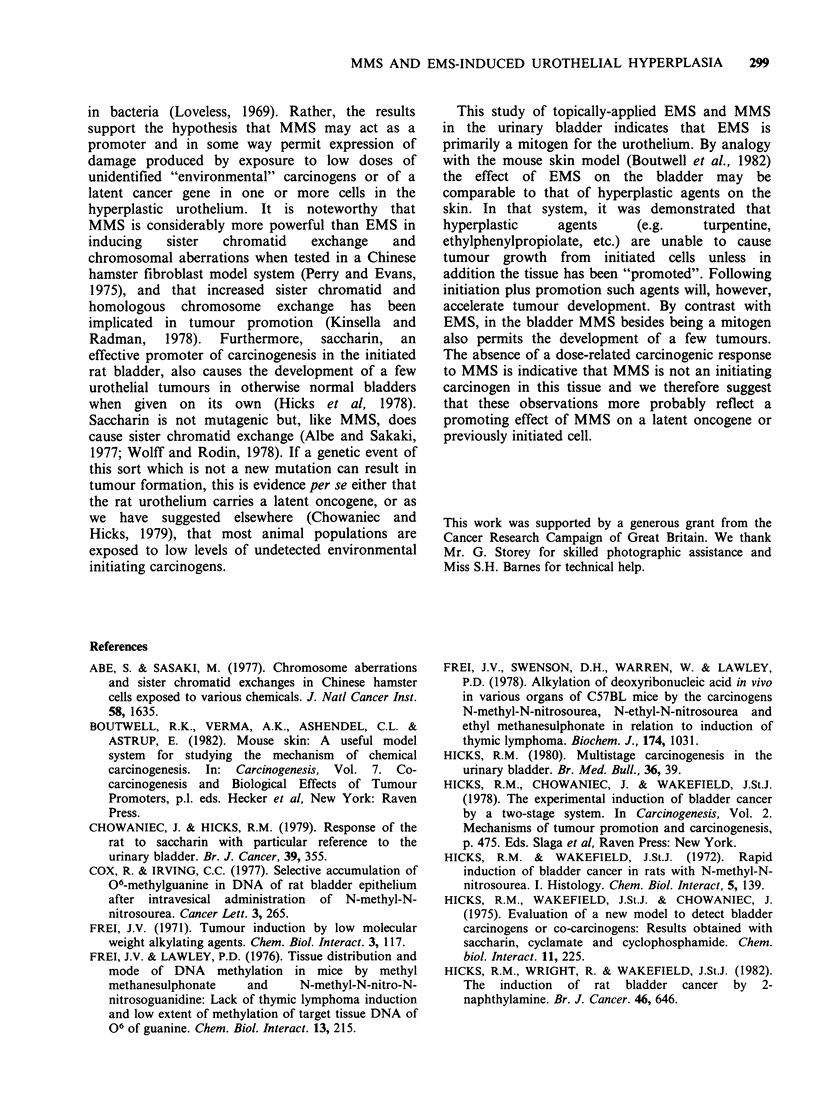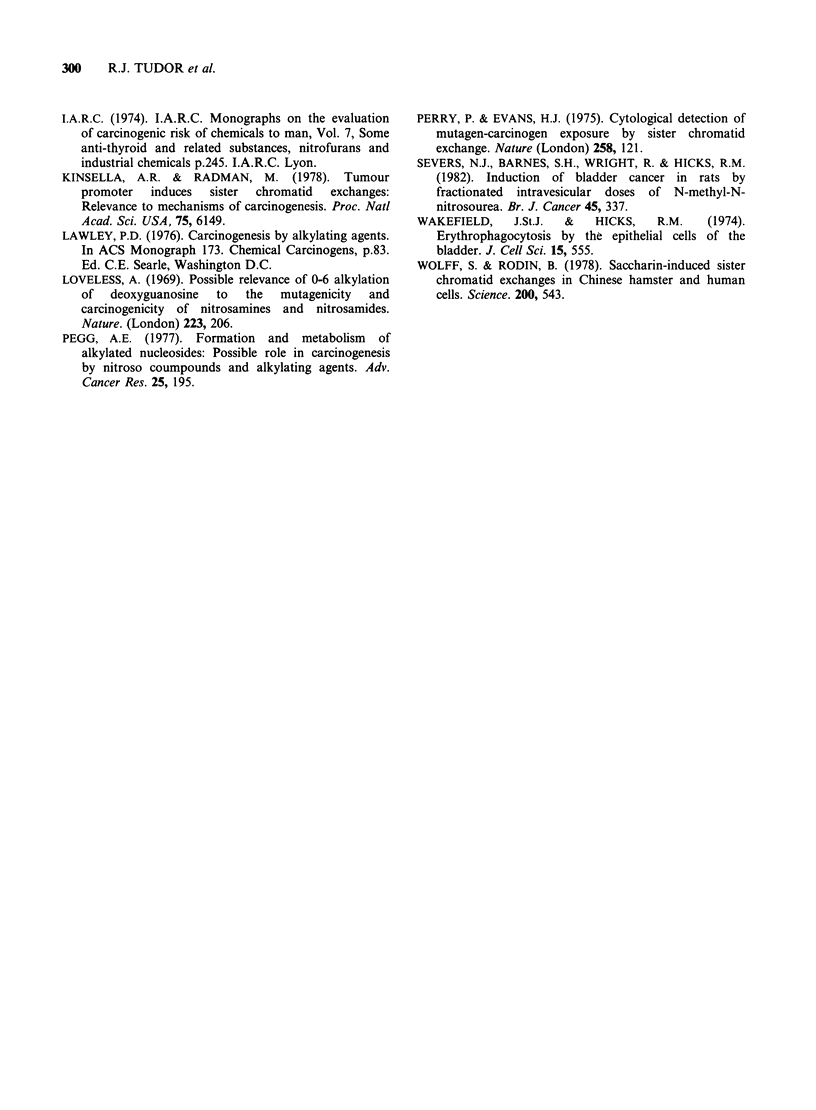# The induction of urothelial hyperplasia by methyl methanesulphonate and ethyl methanesulphonate.

**DOI:** 10.1038/bjc.1983.184

**Published:** 1983-08

**Authors:** R. J. Tudor, N. J. Severs, R. M. Hicks

## Abstract

**Images:**


*Br. J. Cancer* (1983), **48**, 289–300

### The induction of urothelial hyperplasia by methyl methanesulphonate and ethyl methanesulphonate

R.J. Tudor, N.J. Severs* & R.M. Hicks

*School of Pathology, The Middlesex Hospital Medical School, London WIP 7LD.*

**Summary** The early and late morphological changes induced in rat bladder urothelium by intravesicular administration of the alkylating agents methyl methanesulphonate (MMS) and ethyl methanesulphonate (EMS) are described. In the short-term, both compounds produced dose-related toxic damage followed by a regenerative hyperplasia of the urothelium. At any given dose-level, the effects of MMS were more severe than those of EMS. Two years after administration of multiple doses of 2.5 mg MMS or 7.5 mg EMS the majority of animals had dose-related simple urothelial hyperplasias with occasional mild dysplasia. However, in three MMS-treated animals the hyperplasias had progressed to well-differentiated transitional-cell carcinomas. No bladder neoplasms were seen in EMS-treated animals. The urothelial response of the rat to MMS and EMS is discussed with reference to the known chemical reactivity of these compounds. It is concluded that EMS is a mitogen for the urothelium and that the few carcinomas which develop following topical exposure of the bladder to MMS do not necessarily reflect any initiating potential in this compound. Rather it is argued that the results are consistent with MMS acting as a promoter in cells which have either been previously initiated or which carry a latent oncogene.

Previous experiments in this laboratory established that the alkylating agent *N*-methyl-*N*-nitrosourea (MNU) is both a powerful hyperplastic agent and a bladder carcinogen when administered by intravesicular instillation (Hicks and Wakefield, 1972). This finding has provided a useful model system in which the mechanisms of carcinogenesis and the effects of modulators can be explored (Hicks, 1980; Hicks *et al.*,1975, 1978; Severs *et al.*, 1982). Intravesicular instillation of two other alkylating agents, *viz.* methyl methanesulphonate (MMS) and ethyl methanesulphonate (EMS) in the short-term produced toxic damage followed by urothelial hyperplasia (Wakefield & Hicks, 1974).

Both EMS and MMS have been reported to induce neoplasia in mice and rats following s.c. or i.p. injection (data reviewed in IARC, 1974). In rats, the lowest doses reported to produce tumours following a single intraperitoneal injection were $100\,mg\,kg^{-1}$ EMS and $72\,mg\,kg^{-1}$ MMS. EMS produced mainly lung and kidney tumours whereas MMS produced local tumours and tumours of the nervous system. Oral administration of MMS to mice for life was reported to increase the incidence of lung tumours and lymphomas. Although there was no indication that EMS and MMS were carcinogenic for the urinary bladder, the fact that in our previous work they were found to elicit urothelial hyperplasia warrants further investigation. The present study was therefore undertaken to determine the short-term dose-response of the rat bladder to single doses of EMS and MMS, and to assess whether single or multiple doses of these agents have any carcinogenic potential for the urothelium in long-term trials.

*Present address: The Cardiothoracic Institute, 2 Beaumont Street, London W1N 2DX.
Correspondence R. M. Hicks.
Received 10 February 1983; accepted 22 April 1983.

### Materials and methods

### Chemicals

Methyl methanesulphonate (MMS) was obtained from Cambrian Chemicals Ltd., Croydon, Surrey and ethyl methanesulphonate (EMS) from Sigma London Chemical Company Ltd., Poole, Dorset. Both chemicals were used as supplied.

### Animals and diet

Specific-pathogen-free female Wistar rats, free from the bladder parasite, *Trichosomoides crassicauda,* were supplied by A. Tuck and Son Ltd. (Battlesbridge, Essex). They were caged in groups of 6 in rooms kept at 19–22°C with a relative humidity of 50–60% and were maintained on Dixon's standard pelleted 41B diet and tap water *ad libitum.* The animals were 6-8 weeks old at the beginning of treatment and weighed 135–175 g. They were observed daily for signs of ill health; those that became ill and whose condition did not improve were killed and subjected to post-mortem examination.

### Intravesicular administration of agents

Fresh solutions of MMS and EMS were prepared in McIlvaine's citric acid/phosphate buffer (pH 7.0)

© The Macmillan Press Ltd., 1983

prior to each dosing session. Catheters were made from 4 cm lengths of Portex tubing (pp10, Portex Ltd., Hythe, Kent) and sterilized in 70% ethanol. Rats were anaesthetized by i.p. veterinary nembutal (May and Baker Ltd., Dagenham, Essex), and a catheter inserted via the urethra into the bladder of each animal. To minimise any alteration in the concentration of MMS or EMS by dilution with urine in the bladder, micturition was induced by gentle pressure to the lower abdomen.

The MMS and EMS solutions were instilled in a volume of 0.15 $cm^3$ using a graduated syringe with a 30 G needle, which fitted into the end of the catheter. After dosing, the catheter was gently withdrawn from each bladder, and the animals returned to cages where they were kept warm during the recovery period.

*Experimental design*

Three experiments were carried out to investigate the short-term effects of MMS and EMS on the urothelium. In the first, rats each received a single intravesical dose of 15 mg (100 mg $kg^{-1}$) of EMS and were killed at intervals of 2 h, 4 h, 1 day, 4 days and 7 days after treatment. In the second experiment, single doses of 3.6 mg (20 mg $kg^{-1}$) and 8.75 (50 mg $kg^{-1}$) of each compound were given and the rats killed at 1 and 7 days post-dosing. Single doses of 2.4 mg (15 mg $kg^{-1}$) MMS and 6.2 mg (35 mg $kg^{-1}$) EMS were used in the third experiment and the animals killed at 1 and 7 days. These experiments enabled a dose of each compound to be selected which elicited a similar biological effect, namely a moderate hyperplasia with minimal toxic damage, for use in the long-term trial. On the basis of the findings (see Results section) a dose of 2.5 mg was selected for MMS, and 7.5 mg for EMS.

For the long-term study, rats were randomly distributed into 9 groups (A to I). Animals in group A were not treated and were maintained as the control group. Rats in groups B–E received one to four doses of 2.5 mg MMS, and those in groups F–I received one to four doses of 7.5 mg EMS. (See Table I for details.) The multiple doses were administered at intervals of 2 weeks.

*Post-mortem procedures and tissue preparation*

Animals that died during the study were autopsied, unless this was precluded by advanced autolysis or cannibalism. Those found *in extremis* or surviving to 2 years after the initial dose were killed by cervical dislocation and their bladders exposed through an abdominal incision. The urethra was clamped and the bladder gently inflated by injection of 0.5 $cm^3$ of 10% phosphate-buffered formalin (pH 7.4) into the lumen with a fine needle introduced through the dome. Its serosal surface was bathed with the same fixative and after 4 minutes fixation *in situ* the bladder was dissected free.

After removal, the bladder was cut longitudinally into two halves and examined under a dissecting microscope for gross lesions (e.g. thickened areas, tumours and calculi). In bladders of normal macroscopic appearance, one half was further fixed in 10% formalin prior to processing and embedding in paraffin wax. Sections of 4 $\mu$m were cut and stained with haematoxylin and eosin. The other half was cut into 1 $mm^3$ blocks and post-fixed in cold 0.1 M cacodylate-buffered 1% osmium tetroxide. After dehydration at room temperature in graded concentrations of alcohols, the tissue was embedded in Spurr resin. Semi-thin (1 $\mu$m) sections were cut and stained with toluidine blue for high resolution light microscopy. At least three blocks from each bladder were examined by this method to complement the results obtained by conventional histology.

In tumour-bearing bladders, areas of each tumour and of the bladder wall were prepared for both wax and resin embedding. Bladders from animals that were found dead were processed for wax-embedding only.

### Results

*Classification of urothelial lesions*

The typical appearance of normal urothelium from an untreated control animal is shown in Figure 1a. For assessment of both short-term and long-term effects of MMS and EMS, urothelial hyperplasia was defined as urothelium more than 3 cell layers thick. Simple hyperplasias were further sub-divided into mild, moderate and marked categories. Mild hyperplasia was defined as areas 4–6 cell layers thick (Figure 1b), moderate hyperplasia as areas 7–10 cells thick (Figure 1c) and marked hyperplasia as areas >10 cell layers thick (Figure 1d). It was also noted whether the lesions were focal or diffuse in nature, the lesions being regarded as diffuse only when >20% of the epithelium was hyperplastic. Nodular and papillary hyperplasias were recorded as a separate category. For each bladder, the grade of lesion recorded was the most severe observed.

The diagnostic criteria for dysplasia, carcinoma *in situ*, and invasive carcinomas are those currently used in this laboratory and have been published (Hicks *et al.*, 1982).

*Short-term effects of MMS and EMS*

Some degree of toxic damage including urothelial

**Figure 1** Classification of urothelial hyperplasias, (a) normal bladder showing 3 cell thick urothelium; (b) mild hyperplasia; (c) moderate hyperplasia; (d) marked hyperplasia. Toluidine blue-stained semi-thin sections. (a) & (b), ×590; (c) & (d), ×230.

changes, necrosis, inflammation and oedema, followed by a regenerative hyperplasia of the urothelium was evident at all dose levels of MMS and EMS. At any given dose level, the effects of MMS were more severe than those of EMS. The toxicity was dose-related and with higher doses the resulting hyperplasias although delayed, were more marked. There was some variation in the toxic response, both within individual bladders and between individual animals.

In the first short-term experiment, in which MMS and EMS were administered at doses of $100 \mathrm{mg\,kg^{-1}}$ (17.5 mg per animal), toxic damage to the urothelium was visible 2 h after dosing. The intercellular spaces were dilated, some cells were vacuolated and there was focal desquamation of many superficial cells. These effects were more conspicuous at 4 h and by 1 day much of the urothelium had been stripped and some necrosis of the underlying tissues was seen. There was pronounced oedema and inflammation of the submucosa; the blood vessels, initially congested with erythrocytes, subsequently ruptured causing extensive haemorrhage. By 4 days, early focal hyperplasia of the urothelium had developed between remaining necrotic areas. Where haemorrhage had occurred into overlying urothelial tissue, erythrophagocytosis was observed as previously reported (Wakefield & Hicks, 1974). At 7 days, some areas of the bladder were still necrotic and, although the supporting stroma had been partially restored by fibrosis, they were not yet re-epithelialised. In adjacent areas, the regenerative hyperplasia of the urothelium was moderate-to-marked in nature.

In the second short-term experiment, in which the effects of lower doses of MMS and EMS were investigated, the condition of the bladder was examined at 1 and 7 days after treatment. After $50 \mathrm{mg\,kg^{-1}}$ MMS, the toxic damage and resultant urothelial hyperplasia were similar to that observed after a dose of 100 mg kg$^{-1}$. However, at lower doses the severity both of the damage and of the subsequent regenerative hyperplasia was reduced. One day after 20 mg kg$^{-1}$ MMS and 50 mg kg$^{-1}$ EMS, there was a loss of superficial and intermediate cells leaving predominantly 1 cell thick urothelium. In small focal areas, there was complete stripping of the urothelium with necrosis of the underlying stroma, but by 7 days, the urothelium was hyperplastic to a moderate or marked degree.

After 15 mg kg$^{-1}$ MMS, and 20 or 35 mg kg$^{-1}$ EMS, urothelial damage at 1 day was limited to loss of the superficial and some intermediate cells. Complete stripping was rare and if present was confined to small localised areas. At 7 days, the urothelial hyperplasia varied in severity from mild and diffuse after the EMS (Figure 2a), to more moderate and diffuse after the MMS (Figure 2b).

**Figure 2** Typical appearance of the urothelium 7 days after a single dose of 20 mg kg$^{-1}$ EMS (a) and 15 mg kg$^{-1}$ MMS (b). Toluidine blue-stained semi-thin sections. (a) ×190; (b) ×210.

In the short-term studies, gross haematuria was observed 1 day after treatment with doses of 50 mg kg$^{-1}$ or more MMS, and with 100 mg kg$^{-1}$ EMS, but did not occur in animals receiving lower doses of these compounds.

From these 3 short-term trials, it was established that 15 mg kg$^{-1}$ MMS (2.5 mg per animal) and between 35 and 50 mg kg$^{-1}$ EMS (6.2–8.75 mg per animal) were the lowest doses that would reliably elicit moderate hyperplasia without haematuria and extensive or persistent toxic damage. Therefore, for the long-term trial in which multiple doses were to be used, fraction sizes of 2.5 mg MMS and 7.5 mg EMS were selected to give a comparable, relatively uniform hyperplastic response.

*Long-term trial*

Most hyperplasias observed consisted simply of thickened urothelium in which an orderly differentiation from basal to superficial cell layers was retained. Superficial cells were usually flattened, although not necessarily differentiated, but in more severe lesions the superficial cells often appeared undifferentiated. Blood vessels were often conspicuous at the base of the urothelium, occasionally infiltrating into it, particularly in the more severe hyperplasias (see Figure 4). With one exception, nodular and papillary urothelial hyperplasias were only observed in bladders where carcinoma was also detected.

The total incidence of urothelial hyperplasia in animals killed at 2 years was dose-related, both for MMS and EMS (Figure 3 and Table I). The fraction sizes selected, namely 2.5 mg MMS and 7.5 mg EMS, produced a remarkably comparable response in the urothelium, as predicted from the

**Figure 3** Incidence of urothelial hyperplasia in response to single and multiple doses of 2.5 mg MMS (△) and 7.5 mg EMS (●). Untreated control group (□) is shown for the zero dose.

**Table I** Terminal pathology of the urothelium

| Group | Treatment | No. of bladders examined* | *State of urothelium % incidence (absolute number shown in parentheses)* Normal | Hyperplastic Mild | Hyperplastic Moderate | Hyperplastic P/N^Δ | Hyperplastic Total | Neoplastic |
|---|---|---|---|---|---|---|---|---|
| A | None | 80 | 83.5 (66) | 14 (11) | 3.5 (3) | — | 17.5 (14) | — |
| B | 1×2.5 mg MMS | 41 | 51 (21) | 39 (13) | 7 (3) | — | 46 (19) | 3 (1)† |
| C | 2×2.5 mg MMS | 23 | 35 (8) | 35 (6) | 26 (6) | — | 61 (14) | 4 (1)‡ |
| D | 3×2.5 mg MMS | 21 | 14 (3) | 57 (11) | 19 (4) | 5 (1) | 81 (17) | 5 (1)† |
| E | 4×2.5 mg MMS | 16 | 19 (3) | 56 (8) | 25 (4) | — | 81 (13) | — |
| F | 1×7.5 mg EMS | 36 | 56 (20) | 36 (12) | 8 (3) | — | 44 (16) | — |
| G | 2×7.5 mg EMS | 23 | 30 (7) | 48 (9) | 22 (5) | — | 70 (16) | — |
| H | 3×7.5 mg EMS | 14 | 29 (4) | 71 (8) | — | — | 71 (10) | — |
| I | 4×7.5 mg EMS | 15 | 20 (3) | 80 (10) | — | — | 80 (12) | — |

*Bladders were from rats surviving to 2 years after the initial dose plus those killed *in extremis* or found dead on trial

ΔP/N refers to urothelial hyperplasias with a papillary/nodular growth pattern

†Papillary transitional cell carcinoma

‡Papillary carcinoma *in situ*

short-term trials. However, the dose-related response to EMS and MMS was less evident if the hyperplasias were sub-divided into mild and moderate. Thus, moderate hyperplasia was seen in some animals after low but not after high doses of EMS. Similarly, there was no clear dose-related response in the incidence of each category of hyperplasia after MMS; overall the lesions observed were more severe than those seen after EMS treatment. The majority of untreated control bladders had normal urothelial differentiation, although 11 animals had mild and 3 had moderate focal hyperplasia (Table I). Mild dysplasias were occasionally seen in urothelia from the control group, but these were generally associated with concomitant hyperplasia. In animals treated with MMS and EMS, dysplasias were notably more frequent and more severe both in urothelium of normal thickness and in hyperplastic tissue. The dysplasias were characterised by large indented nuclei in the basal and intermediate layers, loss of normal nuclear orientation and occasional large multinucleate intermediate and binucleate basal cells (Figures 5–7).

In one MMS-treated animal, the bladder was lined by a grossly hyperplastic urothelium with a papillary/nodular (P/N) growth pattern. (Figure 8). The urothelium was well differentiated with relatively few areas of dysplasia (Figure 9), and although this was a borderline case, we classified it as a hyperplastic, rather than a neoplastic lesion. This bladder also contained a small free-lying calculus.

Three urothelial carcinomas developed in MMS-treated animals, but none in animals treated with EMS. Two were isolated, large exophytic, well-differentiated transitional-cell carcinomas of the bladder with invasion of the papillary stalk (Pla) (Figure 10). In one of these tumour-bearing bladders there was a small area of squamous metaplasia at some distance from the tumour (Figure 11) and also a small free-lying calculus. The third neoplasm was a carcinoma *in situ* in a ureter proximal to the uretero-vesical junction. In this lesion, the urothelium showed papillary hyperplasia with focal areas of obvious nuclear pleomorphism and disturbance of cellular polarity (Figure 12); mitotic figures were prominent (Figure 13). There was diffuse papillary hyperplasia of the bladder urothelium in the same animal, but no sign of urolithiasis.

### Discussion

The short-term effects described here of instilling either MMS or EMS into the bladder, confirm and

4 5 6 7

**Figures 4–7** Hyperplastic urothelium with blood vessel infiltration from a rat killed 2 years after a single dose of 7.5 mg EMS. (Figure 4). Features of mild urothelial dysplasia; irregular nuclear profiles (arrows, Figure 5), multinucleate cells (arrows, Figure 6) and disorientated nuclei of variable size (arrows, Figure 7). Toluidine blue-stained semi-thin sections. × 580.

**Figure 8** Survey view of a diffuse papillary/nodular hyperplasia from an animal given 3 instillations of 2.5 mg MMS H & E-stained wax section. × 40.

**Figure 9** Semi-thin section showing fine detail of the hyperplastic urothelium from the same bladder as in **Figure 8.** Toluidine blue-stained. × 180.

extend the preliminary observations of toxic damage followed by urothelial hyperplasia reported previously (Wakefield & Hicks, 1974). The present study shows the severity and time-course of the response to both agents to be dose related. The effects of MMS were consistently more severe than those of EMS, and in order to elicit the same degree of moderate hyperplasia with minimal toxic damage, a three-fold dose of EMS was required by comparison with MMS (i.e. 7.5 mg and 2.5 mg respectively). These findings are in accord with the toxic effects produced by systemic administration of EMS and MMS ($LD_{50}$ values of 434 mg $kg^{-1}$ and 180 mg $kg^{-1}$ respectively (Frei, 1971) (see footnote a). In mutagenicity tests also, alkylating agents which react chemically via an $S_N2$ mechanism are more cytotoxic than those reacting via an $S_N1$ mechanism (see footnote b) (Pegg, 1977). MMS is a typical $S_N2$ type agent, whereas EMS shows both $S_N1$ and $S_N2$ characteristics (Lawley, 1976). The cytotoxic and hyperplastic effects of these compounds instilled directly into the bladder thus reflect their direct chemical reactivity.

For the long-term experiments, doses of MMS and EMS were selected which gave a virtually identical dose-response pattern in terms of the total numbers of proliferative lesions produced. In addition to its strong hyperplastic activity MMS, but not EMS, was weakly carcinogenic for the urothelium. Topical exposure of the bladder to

[a]The $LD_{50}$ value of 880 mg $kg^{-1}$ for EMS given in Frei, 1971, was a printer's error. (Personal communication from author).

[b]For $S_N1$ reagents the rate-limiting step is ionisation to a carbonium ion which, being extremely electrophilic, can bind with all nucleophilic centres in the DNA. $S_N2$ reagents do not form an intermediate carbonium ion and the transition complex formed has a relatively low electrophilic reactivity. They therefore tend to react more exclusively at the major nucleophilic centre in DNA, i.e. $N^7$ of guanine.

**Figure 10** One of two large exophytic transitional cell carcinomas observed after MMS treatment (1 × 2.5 mg). H & E-stained wax section. × 16.

MNU leads to formation of both 7-methylguanine and $O^6$-methylguanine in urothelial DNA, but since the enzymatic excision of $O^6$-methylguanine takes place at a slower rate than does the repair of other DNA adducts (Cox & Irving, 1977), it is this particular adduct which persists. There is now considerable evidence to support the hypothesis of Loveless (1969) that $O^6$-alkylation of guanine is an important cause of miscoding in DNA and hence of mutation and tumour induction (reviewed by Lawley, 1976). In experiments where the alkylating agents were administered systemically and a range of organs were examined (e.g. brain, liver and thymus), MMS and EMS by comparison with MNU produced little alkylation at the $O^6$ position of guanine and their major alkylation products were 7-methylguanine and 7-ethylguanine respectively (Frei & Lawley, 1976; Frei et al., 1978). Nevertheless, both MMS and EMS are carcinogenic in a variety of organ systems, although they are far less potent than MNU (IARC, 1974). Working on the assumption that differences in carcinogenic activity may be related to the ability of the agent to alkylate the $O^6$ position of the guanine residue in DNA, Frei et al. (1978), using a thymic lymphoma model, calculated that the ratio of equipotent doses

**Figure 11** An area of squamous metaplasia with keratinization from the same bladder as that bearing the tumour illustrated in **Figure 10.** H & E-stained wax section. ×170.

**Figure 12** Low power view of the ureter in which carcinoma *in situ* was detected. Gross papillary hyperplasia is evident with dysplastic areas. From an animal treated with 2×2.5 mg MMS. H & E-stained wax section. ×160.

**Figure 13** High power view of an area from the ureter featured in **Figure 12.** The urothelium shows increased cellularity with disorientated pleomorphic cells and the presence of abnormal mitoses (arrows). The lesion was classified as carcinoma *in situ.* H & E-stained wax section. ×480.

of EMS and MMS by comparison with MNU would be 21 and 144 respectively. If the induction of bladder cancer by these alkylating agents depends solely on the formation of $O^6$-methylguanine in urothelial DNA, then the data of Frei *et al.* (1978) predict that, as we confirmed, of the three MNU should be the most powerful carcinogen for the urothelium. However, they also predict that EMS should be considerably more carcinogenic than MMS, which is the reverse of what we found. Considered together, these observations indicate that after topical application of these alkylating agents to the urothelium, the carcinogenic response may not depend solely on their ability to alkylate DNA, but may reflect other biological phenomena.

Analysis of the proliferative response of the urothelium reveals some interesting differences in the effect of EMS and MMS on this tissue. With both compounds, the total number of proliferative lesions produced gave excellent dose-related measure of exposure (Figure 4). With EMS, however, none of the hyperplasias showed abnormal growth patterns or gross dysplasia and the more severe lesions were found after the lower rather than after the higher dose levels. These expanded populations of normal urothelial cells represent the response of the urothelium to the cytotoxic damage caused by EMS and the absolute thickness of the urothelium will depend on the balance between the rates of cell death and of cell regeneration. The absence of marked dysplasia, abnormal growth patterns or any other signs of possible neoplastic conversion, implies that whatever the reaction may be between topically-applied EMS and the urothelial target cell DNA, it is not a mutagenic event.

MMS, on the other hand, as well as inducing flat proliferative response also produced four lesions with abnormal growth patterns, either P/N hyperplasia or papillary carcinoma. Admittedly, all were well differentiated and not aggressively invasive, i.e. they were comparable to the relatively benign papillomas produced by promoters acting on skin previously initiated by exposure to a carcinogen. This raises the possibility that MMS, unlike EMS which acts as a mitogen, may have some other specific effect on the urothelial cells. The lack of a clear-cut dose-related carcinogenic response of the urothelium to MMS does not support the theory that MMS is acting as an initiating carcinogen like MNU. It is also consistent with the fact that MMS is the least effective of the three alkylating agents at inducing point mutations in bacteria (Loveless, 1969). Rather, the results support the hypothesis that MMS may act as a promoter and in some way permit expression of damage produced by exposure to low doses of unidentified "environmental" carcinogens or of a latent cancer gene in one or more cells in the hyperplastic urothelium. It is noteworthy that MMS is considerably more powerful than EMS in inducing sister chromatid exchange and chromosomal aberrations when tested in a Chinese hamster fibroblast model system (Perry and Evans, 1975), and that increased sister chromatid and homologous chromosome exchange has been implicated in tumour promotion (Kinsella and Radman, 1978). Furthermore, saccharin, an effective promoter of carcinogenesis in the initiated rat bladder, also causes the development of a few urothelial tumours in otherwise normal bladders when given on its own (Hicks *et al,* 1978). Saccharin is not mutagenic but, like MMS, does cause sister chromatid exchange (Albe and Sakaki, 1977; Wolff and Rodin, 1978). If a genetic event of this sort which is not a new mutation can result in tumour formation, this is evidence *per se* either that the rat urothelium carries a latent oncogene, or as we have suggested elsewhere (Chowaniec and Hicks, 1979), that most animal populations are exposed to low levels of undetected environmental initiating carcinogens.

This study of topically-applied EMS and MMS in the urinary bladder indicates that EMS is primarily a mitogen for the urothelium. By analogy with the mouse skin model (Boutwell *et al.,* 1982) the effect of EMS on the bladder may be comparable to that of hyperplastic agents on the skin. In that system, it was demonstrated that hyperplastic agents (e.g. turpentine, ethylphenylpropiolate, etc.) are unable to cause tumour growth from initiated cells unless in addition the tissue has been "promoted". Following initiation plus promotion such agents will, however, accelerate tumour development. By contrast with EMS, in the bladder MMS besides being a mitogen also permits the development of a few tumours. The absence of a dose-related carcinogenic response to MMS is indicative that MMS is not an initiating carcinogen in this tissue and we therefore suggest that these observations more probably reflect a promoting effect of MMS on a latent oncogene or previously initiated cell.

This work was supported by a generous grant from the Cancer Research Campaign of Great Britain. We thank Mr. G. Storey for skilled photographic assistance and Miss S.H. Barnes for technical help.

### References

ABE, S. & SASAKI, M. (1977). Chromosome aberrations and sister chromatid exchanges in Chinese hamster cells exposed to various chemicals. *J. Natl Cancer Inst.* **58,** 1635.

BOUTWELL, R.K., VERMA, A.K., ASHENDEL, C.L. & ASTRUP, E. (1982). Mouse skin: A useful model system for studying the mechanism of chemical carcinogenesis. In: *Carcinogenesis,* Vol. 7. Co-carcinogenesis and Biological Effects of Tumour Promoters, p.l. eds. Hecker *et al,* New York: Raven Press.

CHOWANIEC, J. & HICKS, R.M. (1979). Response of the rat to saccharin with particular reference to the urinary bladder. *Br. J. Cancer,* **39,** 355.

COX, R. & IRVING, C.C. (1977). Selective accumulation of $O^6$-methylguanine in DNA of rat bladder epithelium after intravesical administration of N-methyl-N-nitrosourea. *Cancer Lett.* **3,** 265.

FREI, J.V. (1971). Tumour induction by low molecular weight alkylating agents. *Chem. Biol. Interact.* **3,** 117.

FREI, J.V. & LAWLEY, P.D. (1976). Tissue distribution and mode of DNA methylation in mice by methyl methanesulphonate and N-methyl-N-nitro-N-nitrosoguanidine: Lack of thymic lymphoma induction and low extent of methylation of target tissue DNA of $O^6$ of guanine. *Chem. Biol. Interact.* **13,** 215.

FREI, J.V., SWENSON, D.H., WARREN, W. & LAWLEY, P.D. (1978). Alkylation of deoxyribonucleic acid *in vivo* in various organs of C57BL mice by the carcinogens N-methyl-N-nitrosourea, N-ethyl-N-nitrosourea and ethyl methanesulphonate in relation to induction of thymic lymphoma. *Biochem. J.,* **174,** 1031.

HICKS, R.M. (1980). Multistage carcinogenesis in the urinary bladder. *Br. Med. Bull.,* **36,** 39.

HICKS, R.M., CHOWANIEC, J. & WAKEFIELD, J.St.J. (1978). The experimental induction of bladder cancer by a two-stage system. In *Carcinogenesis,* Vol. 2. Mechanisms of tumour promotion and carcinogenesis, p. 475. Eds. Slaga *et al,* Raven Press: New York.

HICKS, R.M. & WAKEFIELD, J.St.J. (1972). Rapid induction of bladder cancer in rats with N-methyl-N-nitrosourea. I. Histology. *Chem. Biol. Interact,* **5,** 139.

HICKS, R.M., WAKEFIELD, J.St.J. & CHOWANIEC, J. (1975). Evaluation of a new model to detect bladder carcinogens or co-carcinogens: Results obtained with saccharin, cyclamate and cyclophosphamide. *Chem. biol. Interact.* **11,** 225.

HICKS, R.M., WRIGHT, R. & WAKEFIELD, J.St.J. (1982). The induction of rat bladder cancer by 2-naphthylamine. *Br. J. Cancer.* **46,** 646.

I.A.R.C. (1974). I.A.R.C. Monographs on the evaluation of carcinogenic risk of chemicals to man, Vol. 7, Some anti-thyroid and related substances, nitrofurans and industrial chemicals p.245. I.A.R.C. Lyon.

KINSELLA, A.R. & RADMAN, M. (1978). Tumour promoter induces sister chromatid exchanges: Relevance to mechanisms of carcinogenesis. *Proc. Natl Acad. Sci. USA*, **75**, 6149.

LAWLEY, P.D. (1976). Carcinogenesis by alkylating agents. In ACS Monograph 173. Chemical Carcinogens, p.83. Ed. C.E. Searle, Washington D.C.

LOVELESS, A. (1969). Possible relevance of 0-6 alkylation of deoxyguanosine to the mutagenicity and carcinogenicity of nitrosamines and nitrosamides. *Nature*. (London) **223**, 206.

PEGG, A.E. (1977). Formation and metabolism of alkylated nucleosides: Possible role in carcinogenesis by nitroso coumpounds and alkylating agents. *Adv. Cancer Res*. **25**, 195.

PERRY, P. & EVANS, H.J. (1975). Cytological detection of mutagen-carcinogen exposure by sister chromatid exchange. *Nature* (London) **258**, 121.

SEVERS, N.J., BARNES, S.H., WRIGHT, R. & HICKS, R.M. (1982). Induction of bladder cancer in rats by fractionated intravesicular doses of N-methyl-N-nitrosourea. *Br. J. Cancer* **45**, 337.

WAKEFIELD, J.St.J. & HICKS, R.M. (1974). Erythrophagocytosis by the epithelial cells of the bladder. *J. Cell Sci.* **15**, 555.

WOLFF, S. & RODIN, B. (1978). Saccharin-induced sister chromatid exchanges in Chinese hamster and human cells. *Science*. **200**, 543.